# National drug utilization trend of analgesics in China: an analysis of procurement data at 793 public hospitals from 2013 to 2018

**DOI:** 10.1186/s40545-021-00325-8

**Published:** 2021-05-25

**Authors:** Honghao Shi, Xianwen Chen, Xueli Liu, He Zhu, Fei Yu, Carolina Oi Lam Ung, Wai Sin Chan, Hao Hu, Sheng Han

**Affiliations:** 1 State Key Laboratory of Quality Research in Chinese Medicine, Institute of Chinese Medical Sciences, University of Macau, Macao, China; 2 International Research Center for Medicinal Administration, Peking University, Beijing, China; 3 Peking University First Hospital, Peking University, Beijing, China; 4 Orthopaedic Department, Conde S. Januario General Hospital, Macao, China

**Keywords:** Pain management, Analgesics, Drug utilization, Procurement cost, Dosage utilization, Public hospital

## Abstract

**Background:**

This research aimed to evaluate analgesic utilization in public hospitals from 2013 to 2018 by analyzing the procurement data of 793 hospitals in China.

**Methods:**

This study applied a retrospective observational study by using a database of the Chinese Pharmaceutical Association. The final dataset covers 30 provinces and municipalities in China, with a total of 793 public hospitals with complete procurement records of analgesics from January 2013 to December 2018. Procurement cost and dosage utilization were analyzed through descriptive trend statistics.

**Results:**

From the procurement cost data, analgesics mainly consisted of opioids and non-steroidal anti-inflammatory drugs (NSAIDs), and the annual cost of both types of drugs increased yearly. The 6-year total cost of opioids accounts for 57% (17,800 million CNY), followed by the cost of NSAIDs accounts for 37% (11,400 million CNY). From 2013 to 2018, the annual cost of opioids nearly doubled, while the annual cost of NSAIDs doubled. The total 6-year clinical drug dosage of opioids accounts for 45% (675 million total defined daily dose (DDD)), and the NSAIDs account for 50% (747 million total DDD). From 2013 to 2018, the annual clinical drug dosage of NSAIDs increased by about 0.6 times. The annual clinical drug dosage of opioids has more than doubled. The top three opioid drugs were dezocine injection, remifentanil injection, and sufentanil injection solution. the top three NSAIDs were flurbiprofen injection, parecoxib injection, and celecoxib oral solution.

**Conclusion:**

In China, analgesics utilization increased rapidly at public hospitals from 2013 to 2018. The utilization of analgesics was highly concentrated in NSAIDs and opioids. Within the two types of analgesics, the main analgesics utilization is also highly concentrated, with some highly risky analgesics. The rational guideline for the utilization of analgesics needs to be established with the support of real-world evidence.

## Background

Analgesics are widely used in pain management. There is a wide range of analgesics, which span across many drug classifications. At present, the analgesics used in the clinical setting can be generally divided into five categories: opioids, non-steroidal anti-inflammatory drugs (NSAIDs), non-opioid central nervous analgesia, local anesthesia, and others [1]. Appropriate use of analgesia can not only alleviate the sufferings of the patients, but also benefit the recovery of the diseases, and has significant social and economic benefits [2, 3]. However, due to a lack of clinical guidelines that details the safe and effective use of analgesics in pain management, there are many cases of unreasonable situations in the clinical use of analgesics around the world [4]. For example, a report from the health system of the United States showed that the proportion of opioid abuse in outpatients with long-term use of opioids was as high as 26% [5]. In China, a study in Xi’an Province evaluating the prescriptions for analgesics showed that the proportion of irrational use of analgesics was nearly 15% [6].

In recent years, the Chinese government has been committed to promoting the rational use of drugs in clinical practice and issued a series of policies, including reducing drug proportion in public hospitals year by year, reforming of health insurance payment from single to compound payment, accelerating the implementation of clinical pathway method, piloting and promoting the use of diagnosis-related groups (DRGs), promoting the rational clinical use of drugs, and so on [7, 8]. In the 13th Five Year Plan, the establishment of a comprehensive evaluation system for clinical drugs has been proposed to be accelerated, and analgesics are one of the key tasks [9]. The National Health Commission (NHC) has entrusted various departments with organizing and carrying out a comprehensive evaluation of clinical drug use in various fields and explore the evaluation results as an essential reference for centralized drug purchase and formulation of clinical drug use guidelines [10, 11]. One of the conventional approaches was to analyze the utilization or reimbursement data for drug utility evaluation.

However, most of the current research on analgesics utilization in China was only based on data from a single hospital or a single city or a province level, which with a narrow time interval. Besides, most of them had no comparative study of multiple types of analgesics. Li et al. [12] used 1-year data to analyze the use of analgesics in nine cancer hospitals. Wang [13], Zhi et al. [14], and Li et al. [15] focused on the use of opioids in small-scale, short-term study design. Studies on analgesics consumption by health care institutions at the national level with conducting a comparative study of multiple types of analgesics and a long-time interval are still rare in China.

Thus, this research aimed to evaluate analgesic utilization in public hospitals from 2013 to 2018 by analyzing the procurement data of 793 hospitals in China. This study will help to generate evidence for policymakers to design health policy interventions to promote rational use of analgesics at national level. In addition, it will provide evidence for global cooperation on optimizing analgesics.

## Methods

### Research design and data collection

The research was reviewed and approved by the Ethics Committee of Peking University for the project of the National Natural Science Foundation of China (71603008).

This study applied a retrospective observational research design by investigating the dataset extracted from an existing database. The research data were collected from the China Pharmaceutical Economic Information Network database (CMEI), which is founded and maintained by the Science and Technology Development Center at the Chinese Pharmaceutical Association. The final dataset of this study covered 30 provinces and 793 public hospitals in China from 1st January 2013 to 31st December 2018.

We collected complete procurement records of analgesics, including drug name, drug administration route, dosage form, procurement amount, and procurement quantity. The exclusion criteria included: (1) no active pharmaceutical ingredients (APIs); (2) excluding external dosage forms (nasal drops, eye drops, syrups, and gargles) except patches.

### Analgesics included

All the drugs from the database follow the ATC classification. The classification of analgesics in this study referred to the generic drug names according to the "Consultations of Experts on Pain Management after Surgery in Adults (2017)" and the "Consensus of Experts in Pain Management in General Surgery (2015)" [16, 17]. The generic name of the drug is used to identify the analgesic. The types of analgesics included:

1. Opioids: morphine, oxycodone, hydromorphone, sufentanil, hydrocodone, fentanyl, butorphino, dezocine, pethidine, pentazocine, nalbuphine, buprenorphine, codeine, dihydrocodeine
2. Non-steroidal anti-inflammatory drugs (NSAIDs): ibuprofen, diclofenac, meloxicam, celecoxib, lornoxicam, acetaminophen
3. Non-opioid central analgesics: tramadol
4. Local anesthetic: bupivacaine, levobupivacaine, ropivacaine, chloropropane
5. Others: ketamine, d-ketamine, gabapentin, pregabalin

### Measurement of procurement cost

Firstly, this study retrospectively analyzed the clinical use of analgesics in China in terms of procurement cost. The price at the 793 hospitals’ procurement records of analgesics from 1st January 2013 to 31st December 2018 was used as procurement cost. The cost data were collected from the China Pharmaceutical Economic Information Network database (CMEI), including drug name, drug administration route, dosage form, procurement amount, and procurement quantity. According to the classification of analgesics, the total procurement cost of 6 years, and the changes in annual procurement cost were measured.

### Measurement of dosage utilization

In addition to procurement cost, this study assessed dosage utilization. In this research, we used total DDD to show the yearly quantity of one type analgesics. Total DDD of each type of analgesic means the sum of drug use in that category in 1 year. DDD refers to the average daily dose of an adult drug used for primary treatment purposes. In this study, the DDD of the drugs is based on the value given by the World Health Organization WHO [18] and drug labels or instructions.

$${\text{Total}}\;{\text{DDD}} = {\text{The}}\;{\text{DDD}}\;{\text{of}}\;{\text{each}}\;{\text{type}}\;{\text{of}}\;{\text{analgesic}} = \sum {\left( {{\text{The}}\;{\text{number}}\;{\text{of}}\;{\text{the}}\;{\text{drug}}/{\text{DDD}}\;{\text{of}}\;{\text{each}}\;{\text{drug}}} \right)} .$$

In this study, the Drug Dosage Index (DDI) uses the clinical analgesics dosage in 2013 as the base value and uses the fixed base ratio to represent the annual dosage index of the analgesic. Then further analyzes the changes in the clinical use of the analgesic.

$${\text{One-year Drug Dosage Index}} = {\text{One-year Drug Dosage Index}}/2013\;{\text{Drug Dosage Index}}.$$

### Data analysis

For data analysis, firstly, this study used descriptive statistics to analyze the characteristics of the overall procurement cost and annual cost share of analgesics. Secondly, this study analyzed the procurement cost and annual cost share of the main analgesic types individually. Thirdly, this study analyzed the clinical use of analgesics quantitatively through DDD. The key analgesics in the main categories were further screened by DDD value, and the DDI was analyzed to demonstrate the changes in clinical use of analgesics. Excel. 2017 software was used.

## Results

### Analysis of analgesics procurement cost

#### Overall procurement cost

As shown in Table 1, regarding the overall procurement cost, opioids and NSAIDs were the two main types of analgesics procured by the 793 sample hospitals. The total procurement cost of analgesics in 793 sample hospitals reached 312 billion CNY in 6 years. The total cost of opioids, which is the highest, was 17,814 (57.10%) million CNY. Followed by NSAIDs, the total cost in 6 years was 11,383 (36.48%) million CNY. The cost of other types of analgesics did not exceed 5%. Among the rest, the cost of local anesthetics was 1047 (3.36%) million CNY; the cost of others analgesics was only 531 (1.70%) million CNY; and the cost of non-opioid central analgesics was 425 (1.36%) million CNY.

**Table 1:** Overall procurement cost of analgesics in 793 public hospitals from 2013 to 2018

Analgesics type	Procurement cost (100 million CNY)	Proportion (%)
Opioids	178.14	57.10
NSAIDs	113.83	36.48
Local anesthetics	10.47	3.36
Others	5.31	1.70
Non-opioid central analgesics	4.25	1.36
Total	312.00	100.00

**Fig. 1:**
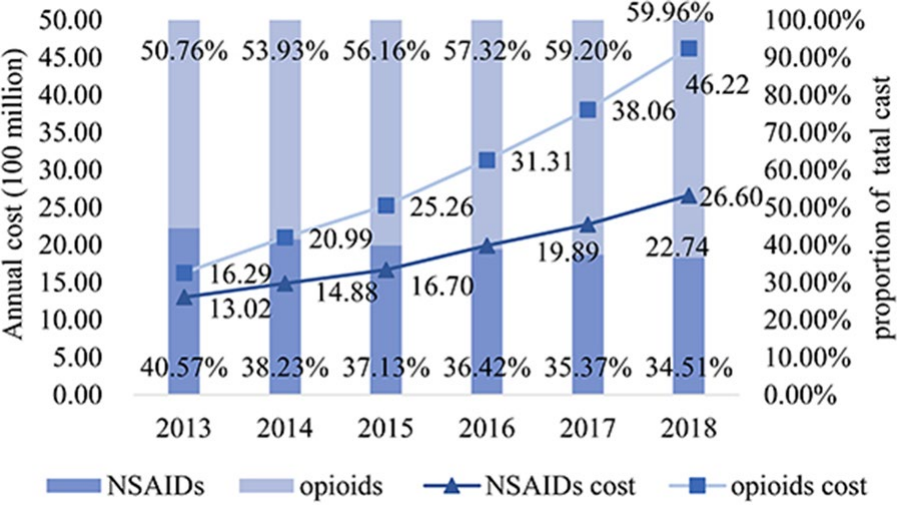
Annual cost and proportion of NSAIDs and opioids from 2013 to 2018 (100 million CNY, %)

The total cost and proportion of opioids and NSAIDs over the past 6 years far exceeded that of other types. The sum of the total cost of the two types of analgesics reached 93.57% of all analgesics. From 2013 to 2018, the annual cost of opioids and NSAIDs has increased yearly, and the increase in opioids is even higher, as shown in Fig. 1.

The annual cost of opioids increased from 1629 million CNY in 2013 to 4622 million CNY in 2018, which was a nearly doubled increase. The annual cost of NSAIDs increased from 1302 million CNY in 2013 to 2660 million CNY in 2018. The proportion of opioids has increased yearly; contrarily NSAIDs have decreased yearly. The proportion of opioids increased from 50.76% in 2013 to 59.96% in 2018, and NSAIDs fell from 40.57% in 2013 to 33.51% in 2018.

#### Procurement cost of opioids

**Fig. 2:**
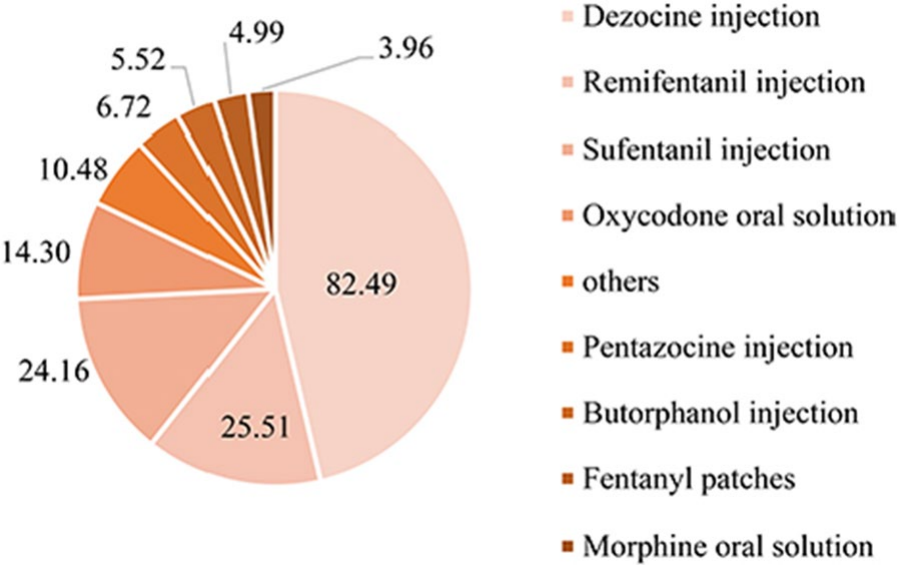
Total cost of main opioids drugs from 2013 to 2018 (100 million CNY)

As shown in Fig. 2, the top 10 opioids drugs included: dezocine injection, remifentanil injection, sufentanil injection, oxycodone oral solution, pentazocine injection, butorphanol injection, fentanyl patches, morphine oral solution, oxycodone injection, and fentanyl injection.

As shown in Fig. 2, the total cost of dezocine injection for 6 years was 8249 (46.31%) million CNY, which was close to half of all opioid analgesics. Followed by remifentanil injection, the total cost was 2551 (14.32%) million CNY. The third one was sufentanil injection, with a total cost of 2416 (13.56%) million CNY. Oxycodone oral agent ranked fourth, with a total cost of 1430 (8.03%) million CNY. The procurement cost of other opioid analgesics accounted for no more than 5%.

**Fig. 3:**
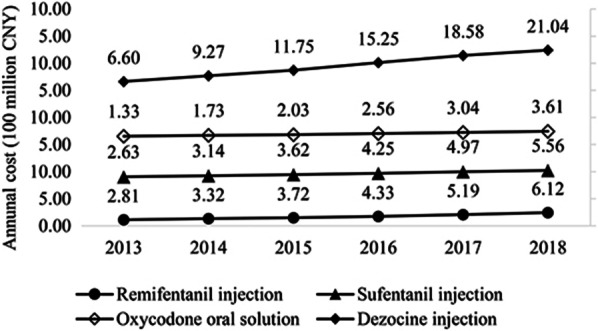
Annual cost of the four main opioid drugs from 2013 to 2018 (100 million CNY)

As shown in Fig. 3, from 2013 to 2018, according to the annual cost data, the average value of the four main opioid drugs showed an increasing trend. Among them, the annual cost of dezocine injection increased the most, rising from 660 million CNY in 2013 to 2104 million CNY in 2018, which was an increase of more than two times. The annual cost of the other three drugs also increased with a relatively flat during the 6 years.

**Fig. 4:**
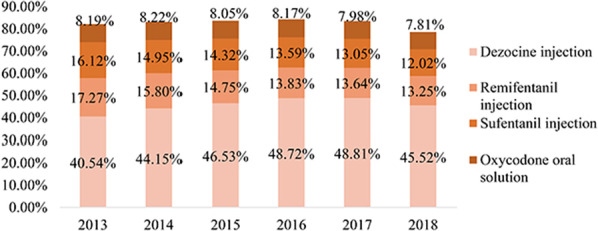
Proportion of four major opioids drugs from 2013 to 2018

As shown in Fig. 4, regarding the cost proportion among the cost of all analgesics, the change trend of the four opioid drugs was different. The annual cost of dezocine injections remained at over 40%, rising yearly from 2013 to 2017, but declined slightly from 2017 to 2018. The cost proportion of remifentanil injection and sufentanil injection showed a slight downward trend. The cost proportion of oxycodone oral solution remained stable.

#### Procurement cost of NSAIDs

As shown in Table 2, the top 10 analgesics of NSAIDs included: flurbiprofen injection, parecoxib injection, celecoxib oral solution, propacetamol injection, flurbiprofen patches, ketorolac injection, ibuprofen oral, diclofenac oral, meloxicam oral, and lornoxicam injection.

**Table 2:** Total cost and proportion of main NSAIDs drugs from 2013 to 2018 (billion CNY, %)

Name of drug	6-year total procurement cost (100 million CNY)	Proportion (%)
Flurbiprofen injection	47.04	41.44
Parecoxib injection	16.79	14.75
Celecoxib oral	13.18	11.58
Propacetamol injection	10.42	9.16
Flurbiprofen patches	9.24	8.11
Ketorolac injection	5.14	4.51
Oral ibuprofen	3.04	2.67
Diclofenac oral	2.81	2.47
Other	6.17	5.42

Five drugs accounted for more than 5% of the NSAIDs 6-year total procurement cost, which included: flurbiprofen injection, parecoxib injection, celecoxib oral solution, propacetamol injection, and flurbiprofen patches. Among them, flurbiprofen injection was the most, with a total cost of 4704 (41.33%) million CNY. The second was parecoxib injection, with a total cost of 1679 (14.75%) billion CNY. Celecoxib oral preparations ranked third, with a total cost of 1318 (11.58%) million CNY; followed by propacetamol injection, with a total cost of 1042 (9.16%) million CNY; flurbiprofen patches cost was 924 (8.11%) million CNY. The varieties that accounted for less than 5% were ranked as ketorolac injection, ibuprofen oral, and diclofenac oral in order of total cost over 6 years.

**Fig. 5:**
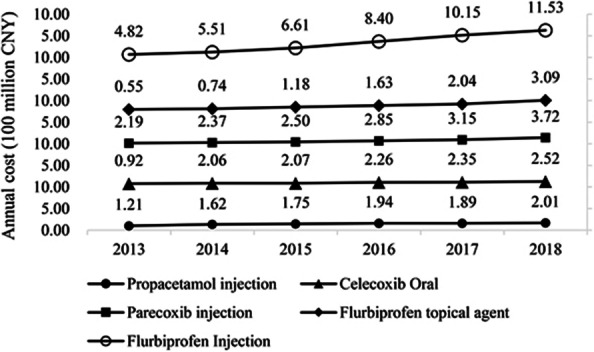
Annual cost of main NSAIDs drugs from 2013 to 2018 (100 million CNY)

As shown in Fig. 5, regarding annual cost, the top five NSAIDs took more than 85% procurement market, and the fees increased year by year. Among them, the annual cost of flurbiprofen injection kept increasing yearly, and the growth rate was the largest. From 2013 to 2018, the annual cost has increased from 482 million CNY to 1153 million CNY, an increase of more than doubled. The annual cost of the other four drugs also increased to a certain extent, with a flat trend.

**Fig. 6:**
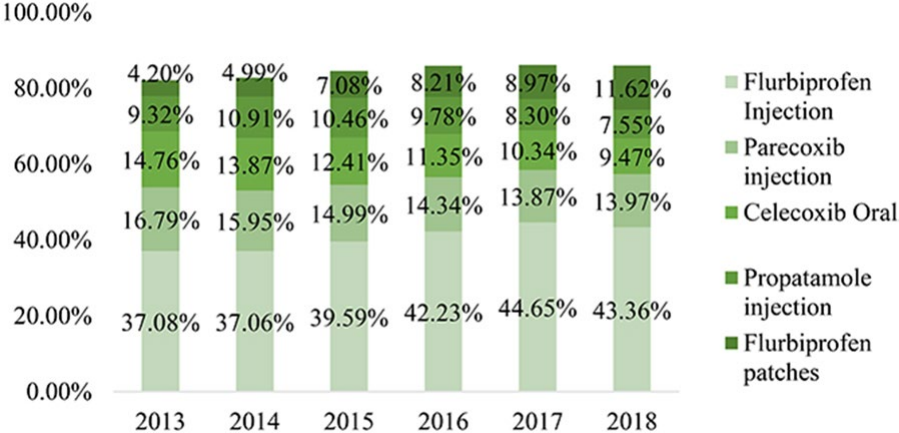
Proportion of major NSAIDs drugs from 2013 to 2018

As shown in Fig. 6, regarding the proportion of annual cost of NSAIDs, the change trend of the five drugs was different. The proportion of flurbiprofen injection remained the highest, up to 44.65% in 2017. Flurbiprofen injections and flurbiprofen patches accounted for an overall upward trend in the annual cost. Parecoxib injections and celecoxib oral administrations showed a general downward trend. Propacetamol injections from 2013 to 2014 rose slightly and gradually declined in the next few years.

### Analysis of clinical dosage utilization

#### Overall dosage utilization

**Fig. 7:**
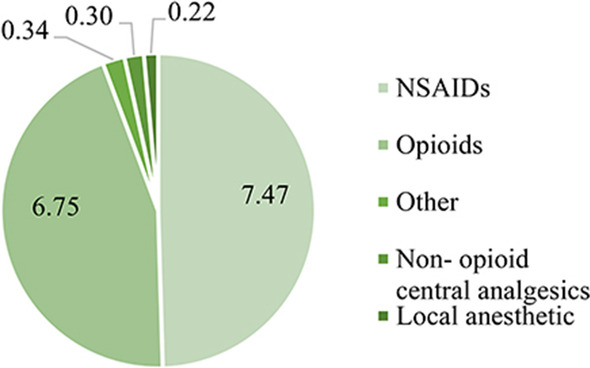
Total clinical dosage utilization of analgesics from 2013 to 2018 (100 million, DDD)

As shown in Fig. 7, regarding the clinical dosage utilization of analgesics, similar to the procurement results, the clinical dosage utilization of NSAIDs and opioids were also the most. The clinical dosage utilization of NSAIDs was the largest. The total dosage for 6 years reached 7.47 (49.54%) billion DDD. The total dosage of opioids used for 6 years was 675 (44.76%) million DDD. The proportion of other types of analgesics did not exceed 5%. Other analgesics dosage was 34 (2.26%) million DDD. The non-opioid central analgesics, the total dosage was 30 (2.01%) million DDD; and the total dosage of local anesthetics was 22 (1.43%) million DDD.

As shown in Table 3, the 6-year total dosage and proportion of opioids and NSAIDs far exceeded that of other types of analgesics, reached 94.30%. From 2013 to 2018, the annual dosage of NSAIDs and opioids showed an upward trend yearly.

**Table 3:** Annual dosage and proportion of opioids and NSAIDs from 2013 to 2018

Annual dosage	2013y	2014y	2015y	2016y	2017y	2018y
Opioids						
Total DDD (100 million)	0.76	0.88	0.99	1.15	1.36	1.61
Proportion^a^	40.69%	41.87%	43.46%	45.05%	47.02%	47.55%
NSAIDs						
Total DDD (100 million)	0.99	1.10	1.15	1.26	1.38	1.60
Proportion^a^	52.98%	51.98%	50.70%	49.26%	47.63%	47.19%

^a^As a percentage of total analgesic annual dosage

The annual total DDD of opioids increased from 76 million in 2013 to 161 million in 2018, an increase of more than doubled. The annual DDD of NSAIDs drugs increased from 99 million in 2013 to 160 million in 2018, an increase of about 0.6 times. The proportion of opioids increased yearly, while NSAIDs decreased yearly. The proportion of opioids increased from 40.69% in 2013 to 47.55% in 2018, and NSAIDs fell from 52.98% in 2013 to 47.19% in 2018.

#### DDI analysis of NSAIDs drugs

**Fig. 8:**
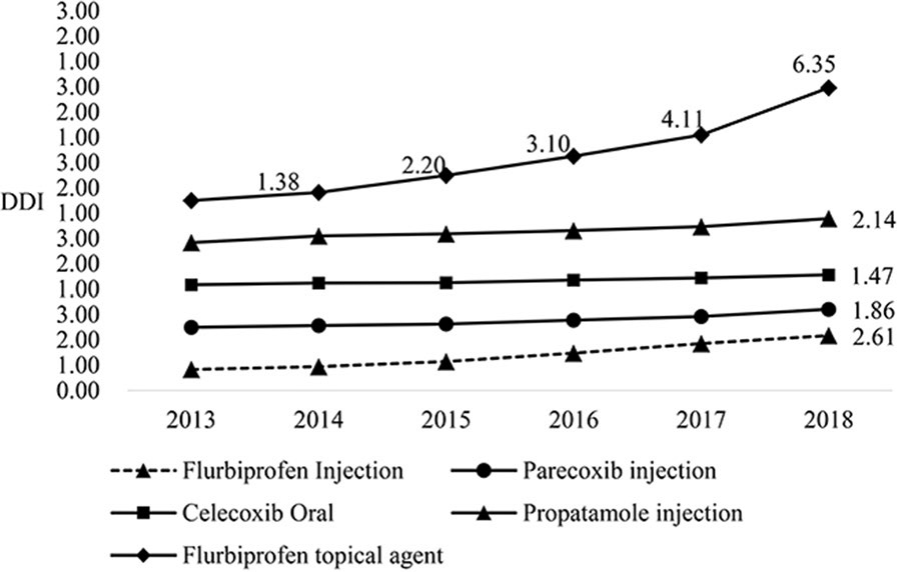
DDI of NSAIDs from 2013 to 2018

The five NSAID drugs that individually accounted for more than 5% of the total procurement cost in 6 years were analyzed in terms of Drug Dosage Index (DDI), as shown in Fig. 8.

The DDI of the five NSAIDs drugs showed an upward trend in the past 6 years. Among them, the increase in the DDI of flurbiprofen patches using was much more significant than that of other drugs. From 2013 to 2018, the DDI increased from 1 to 6.35, and the dosage increased by about five times. The growth trend of the dosage of the other four NSAIDs drugs was relatively flat, besides the growth rate was relatively close.

#### DDI analysis of opioids drugs

**Fig. 9:**
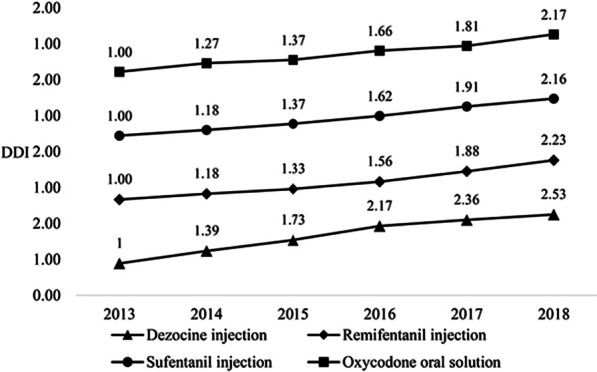
DDI of opioids from 2013 to 2018

Four opioid analgesics individually accounted for more than 5% of the total cost in 6 years, including dezocine injection, remifentanil injection, sufentanil injection, and oxycodone oral solution. The DDI change of these four drugs had been analyzed, as shown in Fig. 9.

The DDI of the four opioid drugs showed an upward trend in the past 6 years. Dezocine injection had a tremendous growth rate, which dosage index in 2018 was 2.53. The increase of remifentanil injection, sufentanil injection, and oxycodone oral agent were relatively close by 1–1.5 times.

## Discussion

The results of procurement cost showed that the analgesics procured by the 793 public hospitals in China during 2013–2018 were mainly opioids and NSAIDs, and the annual procurement cost for these two types analgesics was  steadily increasing. Among opioid analgesic, dezocine injection took up the largest proportion in terms of procurement cost and dosage utilization. Similarly, among the NSAIDs, flurbiprofen injection ranked the highest by these two measurements. These findings suggest that dezocine was becoming increasing dominant for relieving pain, and flurbiprofen was the NSAIDs of choice when it came to pain management in China. Further discussion about these findings in consideration of the pharmacological and economic nature of these medications is as follows.

Dezocine is an opioid analgesic with both agonist and antagonist activity. It is equipotent with morphine and has been used as a post-operative and cancer analgesic but its distribution was discontinued in the US in 1999 [19, 20]. However, as reconfirmed in this study, dezocine currently is still commonly used in China for moderate-to-severe pain and occupied over 45% of the nation’s opioid analgesics market [21]. This research found that dezocine was the analgesics with largest procurement cost and DDD. Previous research results showed that the incidence of clinical adverse reactions of dezocine was higher than other analgesics. For instance, clinical use of dezocine would prolong the hospital stay of patients [22]. Finucane et al. also reported that high-dose dezocine had lower analgesic effects than other low-dose analgesics [23]. However, some studies in China had shown that dezocine could be more effectively used for pain management. It was recommended for clinical use from its strong analgesic effect [24], weak respiratory depression [25], and good quality of patient recovery [26]. Earlier studies also suggested that dezocine had a safe and tolerable side-effects profile, including lack of physical dependence capacity and limited respiratory depression [27, 28]. Thus, considering the high usage and the mixed reports about the safety profile of this medication, further investigation is needed to evaluate the clinical value of dezocine, results of which can be used to inform the development of practice guidelines.

The wide use of dezocine may also be explained by the impact of insurance system as medical insurance has a huge impact on the clinical use of analgesics. An Australian study conducted by Schaffer et al. reported that the subsidy of oxycodone/naloxone-containing medication was associated with a significant increase in the growth rate of dispensing volume [29]. The high use volume of dezocine injection in China may also exemplifies the positive effect of subsidy on the uptake of a medication. Such widely accepted affordability might have contributed to the dominant share of dezocine injection across the analgesic medications. Before 2018, dezocine injection was enlisted and subject to reimbursement under the National Medical Insurance scheme in China. This meant that patients had to pay minimal fee only for dezocine injection. However, the market share of dezocine injection decreased from 48.81% in 2017 to 45.52% in 2018. Furthermore, dezocine was removed from the National Medical Insurance List in 2019. The impact of reimbursement policy change on the clinical utilization of dezocine needs further investigation.

Opioid analgesics are commonly prescribed for pain in China as shown in this study and across other countries. For instance, in the United States, in 2012 health care providers wrote 259 million prescriptions for opioid pain medication, enough for every adult in the United States to some form of opioid analgesic for pain management [30]. Nevertheless, the use of opioid pain medication is associated with serious risks, including overdose and opioid use disorder, which might also lead to death cases [31–33]. For instance, research results had repeatedly reported the adverse reactions [34] and potential risks of drug addiction [35] of the fentanyl group of opioid analgesics. As shown in this study, not only that dezocine injection was often used for pain in China, other opioid analgesics such as sufentanil and remifentanil were also commonly prescribed to relieve pain in China. While opioid analgesics are effective pain medications, the selection should be carefully assessed based on a number of pharmacologic factors and patient variable, important considerations of the “power” of opioids, the class and the associated differential toxicities, duration of effects and other pharmacokinetics factors should be taken into account on the decision-making process [36, 37].

Further to the concerns about opioid prescribing, it has been shown that opioid drugs prescribed by surgeons to treat acute pain following surgery might remain unused [38], which results in excess medication in the community available for diversion [39]. Identifying strategies to reduce irrational use of opioid medications could mitigate the unnecessary risks to the patients. Considering there is a lack of consensus on the prescribing standards about opioid prescribe and wide variation in opioid prescribing practice existed among physicians practicing even within the same department [40], it is important to develop authoritative guidelines on prescribing opioids for different types of pain [41]. For instance, in 2016 the Centers for Disease Control and Prevention (CDC) in the US released a “Guideline for Prescribing Opioids for Chronic Pain” [42], which had been widely adopted to help improve a safer, more effective treatment course that involves opioid medications [43, 44].

The high use volume of NSAIDs in China is also alarming as NSAIDs are linked to many and sometimes serious adverse drug reactions such as kidney injury, gastrointestinal effects, and cardiovascular events [45–47]. NSAIDs are associated with acute kidney injury and may contribute to further disease progression in patients with chronic kidney disease [48, 49]. NSAIDs-related gastrointestinal complications might range from mild-to-severe dyspeptic symptoms, development of gastric or duodenal ulceration, hemorrhage or perforation and other events potentially leading to hospitalization or death [50]. At the same time, all NSAIDs were also found to be associated with an increased risk of acute myocardial infarction [51–53]. Rimon et al. [54] further reported that celecoxib had a high risk of thrombosis. Risk of myocardial infarction with celecoxib was comparable to that of traditional NSAIDS, and such risk was greatest during the first month of NSAIDs use and with higher doses [55].

In this study, NSAIDs was the most common medication group for pain management after opioid analgesics in China and the top three most common NSAIDS in terms of procurement cost and dosage were flurbiprofen injection, parecoxib injection, and celecoxib oral solution. Similar high use volume of NSAIDs has also been observed in other countries. In European countries, such as France, the top three drugs of NSAIDs were ketoprofen, ibuprofen, and piroxicam [56], while in the Middle Eastern countries, diclofenac and ibuprofen are mainly used [57]. Despite growing attention to the dangers of NSAIDs use especially among high-risk populations, use is common among patients with predisposing factors for NSAIDs-related ADR [58]. Clinically, assessment of the absolute risks regarding kidney, gastrointestinal and cardiovascular complications needs to take into account when deciding on the use and choice of NSAIDs [58]. Besides, pharmacovigilance measures specific to these NSAIDs should be strengthened to monitor possible risks and capture all the necessary evidence to inform a standard approach with regard to the use of NSAIDs in the country.

In summary, the utilization composition of analgesics was varied from other countries with the increaseing utilization quantity for anlagesics in China, and widely using of some highly risky analgesics either requires special attention. Therefore, two suggestions were given including: (1) Analgesics utilization clinical decision-making should be supported by strong clinical evidence, owing to the lack of enough guidelines for analgesics use in China. Health Information System for the use of analgesics should be strengthened to collect and monitor real-world evidence and provide a basis for establishing analgesics guidelines. (2) Health policies for analgesic regulation need to be designed and implemented with strong government administration. Notably, the analgesics procurement can be improved by authoritative clinical and economic evaluation evidence.

### Research strength and limitations

This study analyzed the clinical usage of analgesics through long-term procurement data from 793 public hospitals in China. According to our knowledge, this is the first study reporting analgesics utilization at the national level in China. Nevertheless, there are still some research limitations that may be addressed in future studies. First, this study analyzed the national analgesics utilization without considering the background of hospitals. Future studies can analyze the impact of the different characteristics of hospitals on analgesics utilization, especially considering the effect of social insurance. Second, this study has not explored the analgesics utilization in a specific disease or patient group or subdivided dosage forms due to data limits. In this study, total DDD is used to analyze the changes in the dosage of drugs, without distinguishing the route of drug administration. There may be some bias in the comparison of total DDD values for different routes of administration. Future studies can collect comprehensive clinical data for critical diseases to examine the real-world utilization of analgesics and their outcomes to institute instructive clinical guidelines.

## Conclusion

In China, analgesics utilization increased rapidly at public hospitals from 2013 to 2018. Opioids and NSAIDs were the main types of analgesics procured by public hospitals at a national level. The top five drugs covered over 85% of the entire analgesic utilization. The composition of analgesic utilization is different from other nations. Guidelines for the clinical use of analgesics in China are required to support the appropriate use of analgesics.

## Data Availability

The data used to support the findings of this study are available from the corresponding author upon reasonable request.

## Abbreviations

API: Active pharmaceutical ingredient
CDC: Disease Control and Prevention
DDD: Defined daily dose
DDI: Drug Dosage Index
DRGs: Diagnosis-related groups
NHC: National Health Commission
NSAIDs: Nonsteroidal anti-inflammatory drugs

## References

[1] Barkin RL (2013). The pharmacology of topical analgesics. J Postgrad Med.

[2] Institute of Medicine (US) Committee on Advancing Pain Research, Care and Education (2012). Relieving pain in America: a blueprint for transforming prevention, care, education, and research.

[3] McCarberg BH, Nicholson BD, Todd KH, Palmer T, Penles L (2008). The impact of pain on quality of life and the unmet needs of pain management: results from pain sufferers and physicians participating in an Internet survey. Am J Ther.

[4] Gordon DB, de Leon-Casasola OA, Wu CL, Sluka KA, Brennan TJ, Chou R (2016). Research gaps in practice guidelines for acute postoperative pain management in adults: findings from a review of the evidence for an American Pain Society Clinical Practice Guideline. J Pain.

[5] Kaye AD, Jones MR, Kaye AM (2017). Prescription opioid abuse in chronic pain: an updated review of opioid abuse predictors and strategies to curb opioid abuse: part 1. J Pain Physician.

[6] Gong XP, Li J, Wang C (2015). Analysis of non-opioid analgesics prescriptions for cancer patients in outpatient oncology. China Pharm.

[7] NHC. Opinions on controlling the unreasonable increase of medical expenses in Public Hospitals. 2015. (**Chinese**).

[8] The State Council. Opinions on further reforming and perfecting the policy of drug production, circulation and use. 2017. (**Chinese**).

[9] The State Council. “Thirteenth Five-Year Plan" for deepening the reform of healthcare. 2016. (**Chinese**).

[10] NHC. National Center for Comprehensive Evaluation of Drug and Health Technology established. 2018. (**Chinese**).

[11] NHC. Notice on drug use monitoring and clinical comprehensive evaluation. 2019. (**Chinese**).

[12] Li R, Zhang YH (2012). Analysis on the utilization of analgesic drugs in 9 cancer hospitals of China in 2012. Chin J Hosp Pharm.

[13] Wang JH. Analysis on the utilization of narcotic analgesics in the inpatients of Gansu Provincial Hospital of Maternal and Child Health from 2006 to 2010. Evaluation and analysis of drug-use in hospitals of China. 2011. (**Chinese**).

[14] Zhi MJ, Wei XM, Gao X (2018). Analysis of the clinical use of opioid analgesics in China. Chin J Pharmacoepidemiol.

[15] Li XP, Qin X, Jing FB, Han B, Sui ZG (2016). Analysis of the utilization of opioid analgesic drugs in 29 Hospitals of Qingdao District. J China Pharm.

[16] Chinese Association of Anesthesiology. Expert consensus on postoperative pain management in adults. J Clin Anesth. 2017. (**Chinese**).

[17] Leng X, Wei J, Liu L (2015). Expert consensus on perioperative pain management in general surgery. J Chin J Gen Surg.

[18] WHO Collaborating Centre for Drug Statistics Methodology. Guidelines for ATC classification and DDD assignment 2020. Oslo; 2019.

[19] Kevin Bylund. Dezocine. xpharm the comprehensive pharmacology reference. 2007;1–5.

[20] Liu RY, Huang XY, Yeliseev A, Xi J, Roth BL (2014). Novel molecular targets of dezocine and their clinical implications. Anesthesiology.

[21] Wang YH, Chai JR, Xu XJ (2018). Pharmacological characterization of dezocine, a potent analgesic acting as a κ partial agonist and μ partial agonist. Sci Rep.

[22] Ding Y, White PF (1992). Comparative effects of ketorolac, dezocine, and fentanyl as adjuvants during outpatient anesthesia. Anesth Analg.

[23] Finucane BT, Floyd JB, Petro DJ (2012). Postoperative pain relief: a double-blind comparison of dezocine, butorphanol, and placebo. South Med J.

[24] Li ZS, Dong H (2012). Research of dezocine in clinical application. Med Recapitul.

[25] Han XP, Huang YL, Sun ZT, Yang YJ, Ma FL (2011). The effects of equivalent dezocine or fentanyl for postoperative analgesia in thyroid patients. J Clin Anesthesiol.

[26] Song SY, Yue XQ (2012). Clinical observation of dezocine in preventing restlessness of patients during general anesthesia. J Pract Med.

[27] Gal TJ, DiFazio CA (1984). Ventilatory and analgesic effects of dezocine in humans. Anesthesiology.

[28] Romagnoli A, Keats AS (1984). Ceiling respiratory depression by dezocine. Clin Pharmacol Ther.

[29] Schaffer AL, Karanges EA, Buckley NA (2019). Increases in controlled-release oxycodone utilisation following the subsidy of oxycodone with naloxone formulations: an Australian population-based study. Pharmacoepidemiol Drug Saf.

[30] Paulozzi LJ, Mack KA, Hockenberry JM (2014). Vital signs: variation among states in prescribing of opioid pain relievers and benzodiazepines—United States, 2012. MMWR Morb Mortal Wkly Rep.

[31] Substance Abuse and Mental Health Services Administration (2013). The DAWN report: highlights of the 2011 Drug Abuse Warning Network (DAWN) findings on drug-related emergency department visits.

[32] Edlund MJ, Martin BC, Russo JE, DeVries A, Braden JB, Sullivan MD (2014). The role of opioid prescription in incident opioid abuse and dependence among individuals with chronic noncancer pain: the role of opioid prescription. Clin J Pain.

[33] Bohnert AS, Valenstein M, Bair MJ (2011). Association between opioid prescribing patterns and opioid overdose-related deaths. JAMA.

[34] Mars SG, Rosenblum D, Ciccarone D (2019). Fentanyl: the many challenges ahead. Addiction.

[35] Mounteney J, Griggiths P, Sedefov R, Evans-Brown M (2019). Fentanils: a serious threat to public health. Addiction.

[36] Steven D, Waldman MD. CHAPTER 344-Opioid Analgesics. In: Pain review. 2009. pp. 635–40.

[37] Mathias L, McDonald D, Sargent W, Shoemaker-Hunt S, Losby J. Improving Opioid prescribing through the development and evaluation of Clinical Decision Support tools across four large healthcare systems. APHA's 2019 annual meeting and expo. 2019. Nov 2–6.

[38] Hill MV, McMahon ML, Stucke RS, Barth RJ (2017). Wide variation and excessive dosage of opioid prescriptions for common general surgical procedures. Ann Surg.

[39] Harris K, Curtis J, Larsen B (2013). Opioid pain medication use after dermatologic surgery: a prospective observational study of 212 dermatologic surgery patients. JAMA Dermatol.

[40] Barnett ML, Olenski AR, Jena AB (2017). Opioid-prescribing patterns of emergency physicians and risk of long-term use. N Engl J Med.

[41] Howard R, Waljee J, Brummett C, Englesbe M, Lee J (2018). Reduction in opioid prescribing through evidence-based prescribing guidelines. JAMA Surg.

[42] Dowell D, Haegerich TM, Chou R (2016). CDC guideline for prescribing opioids for chronic pain—United States, 2016. MMWR Recomm Rep.

[43] Cross AJ, Buchbinder R, Bourne A (2019). Barriers and enablers to monitoring and deprescribing opioid analgesics for chronic non-cancer pain: protocol for a qualitative evidence synthesis using the Theoretical Domains Framework. BMJ Open.

[44] Schatz AA, Oliver TK, Swarm RA (2020). Bridging the gap among clinical practice guidelines for pain management in cancer and sickle cell disease. J Natl Compr Cancer Netw.

[45] Leonard CE, Freeman CP, Newcomb CW (2012). Proton pump inhibitors and traditional nonsteroidal anti-inflammatory drugs and the risk of acute interstitial nephritis and acute kidney injury. Pharmacoepidemiol Drug Saf.

[46] Gooch K, Culleton BF, Manns BJ (2007). NSAID use and progression of chronic kidney disease. Am J Med.

[47] Bhala N, Emberson J, Merhi A (2013). Vascular and upper gastrointestinal effects of non-steroidal anti-inflammatory drugs: meta-analyses of individual participant data from randomised trials. Lancet.

[48] Zhang XY, Donnan PT, Bell S, Guthrie B (2017). Non-steroidal anti-inflammatory drug induced acute kidney injury in the community dwelling general population and people with chronic kidney disease: systematic review and meta-analysis. BMC Nephrol.

[49] Lipworth L, Abdel-Kader K, Morse J (2016). High prevalence of non-steroidal anti-inflammatory drug use among acute kidney injury survivors in the southern community cohort study. BMC Nephrol.

[50] Brune K, Patrignani P (2015). New insights into the use of currently available non-steroidal anti-inflammatory drugs. J Pain Res.

[51] Sharma M, Gulmez SE, Lassalle R, Moore N (2012). Use of NSAIDs in France: analysis from French reimbursement (CNAM-TS) database. J Pharm Res.

[52] Inotai A, Hanko B, Meszaros A (2010). Trends in the non-steroidal anti-inflammatory drug market in six Central-Eastern European countries based on retail information. Pharmacoepidemiol Drug Saf.

[53] Meagher EA (2004). Cardiovascular and renovascular implications of COX-2 inhibition. Curr Pharm Des.

[54] Nussmeier NA, Whelton AA, Brown MT (2005). Complications of the COX-2 inhibitors parecoxib and valdecoxib after cardiac surgery. N Engl J Med.

[55] Bally M, Dendukuri N, Rich B (2017). Risk of acute myocardial infarction with NSAIDs in real world use: bayesian meta-analysis of individual patient data. BMJ.

[56] Kuffner EK, Green JL, Bogdan GM (2007). The effect of acetaminophen (four grams a day for three consecutive days) on hepatic tests in alcoholic patients—a multicenter randomized study. BMC Med.

[57] Rimon G, Sidhu RS, Lauver DA (2010). COXIBs interfere with the action of aspirin by binding tightly to one monomer of cyclooxygenase-1. Proc Natl Acad Sci USA.

[58] Adams RJ, Appleton SL, Gill TK, Taylor AW, Wilson DH, Hill CL (2011). Cause for concern in the use of non-steroidal anti-inflammatory medications in the community-a population-based study. BMC Fam Pract.

